# Uncovering yield-related traits loci in Chinese wheat landraces using GWAS

**DOI:** 10.3389/fpls.2026.1838087

**Published:** 2026-06-15

**Authors:** Ying Guo, Zhe Zhang, Rui He, Rui Ma, Fangping Yang, Lijun Zhang, Zongbing Zhan, Na Liu, Caixia Lan, Bin Bai

**Affiliations:** 1 Wheat Research Institute, Gansu Academy of Agricultural Sciences, Lanzhou, China; 2 Hubei Hongshan Laboratory, National Key Laboratory of Crop Genetic Improvement, College of Plant Science and Technology, Huazhong Agricultural University, Wuhan, Hubei, China

**Keywords:** candidate gene, elite haplotype, genome-wide association analysis, spikelet trait, thousand-kernel weight, wheat landrace

## Abstract

The genetic basis of wheat yield is highly complex, being collectively influenced by key traits such as plant morphology, spike structure, and grain morphology. Therefore, exploring the genetic regulatory mechanisms underlying yield-related traits is crucial for breeding high-yield wheat varieties. In the present study, a panel of 372 wheat landraces from Gansu province, China, was phenotyped four yield-related traits: spike length (SL), spikelet number per spike (SNS), grain number per spike (GNS), and thousand−kernel weight (TKW) over four years across five field environments at two locations (Qingshui and Lanzhou) in Gansu province. Genotyping was performed using a 16K SNP array, and genome-wide association studies (GWAS) were conducted on the multi-environment phenotypic data employing the Bayesian-information and Linkage-disequilibrium Iteratively Nested Keyway (BLINK), Compressed Mixed Linear Model (CMLM), Fixed and Random Model Circulating Probability Unification (FarmCPU), Mixed Linear Model (MLM) and Multi-Locus Mixed Model (MLMM) models. In total, we identified nine loci for SL, four loci for GNS, one loci for SNS, and five loci for TKW. The phenotypic variation explained (PVE) by these loci ranged from 2.0% to 22.0%. Each locus exhibits 2–4 haplotypes and showed significant differences between haplotypes. Furthermore, 16 candidate genes associated with panicle traits were predicted and they encoded proteins such as squamosa promoter-binding protein, F-box family protein, ferredoxin-NADP-oxidoreductase 2, aspartic proteinase Asp1, Pre-mRNA splicing factor and so on. These results provide novel genetic loci for wheat yield improvement, offering valuable resources for future breeding programs.

## 1 Introduction

Yield has always been a core objective in crop breeding. As a global staple crop, enhancing wheat yield potential is crucial for alleviating food security pressures (Ding et al., 2024). From 1961 to 2022, wheat yield per unit area in China increased from 559.1 kg/ha to 5855.2 kg/ha, an approximately 10.47-fold increase with an average annual compound growth rate of 3.93%. However, the yield growth rate has recently slowed significantly, alongside increased inter-annual fluctuations. For instance, the yield per unit area in 2023 decreased by approximately 1.3% compared to 2022 (FAO, 2023). This slowdown may be closely associated with frequent extreme climate events, intensified biotic and abiotic stresses, and increased genetic homogeneity among major cultivated varieties.

Wheat yield is primarily determined by three key components: spike number per unit area, grain number per spike (GNS), and thousand−kernel weight (TKW). The synergistic improvement of these components is a crucial strategy for yield enhancement (Evans, 1993). Regarding morphological traits, spike length, as a core characteristic of spike architecture, directly affects spikelet density, grain number per spike, and thousand−kernel weight (Guo et al., 2018). Therefore, elucidating the genetic basis of yield−related traits is of significant practical importance for stabilizing and further increasing wheat yield.

Identifying genes or genetic loci that control key yield traits such as TKW, GNS, and spikelet number per spike (SNS) is a central strategy for improving wheat yield. Most of these quantitative traits are regulated by multiple genes in a coordinated manner. To date, several genes or transcription factors associated with TKW have been identified, such as *TaGW2*, *TaGDSL-7D*, *TabHLH95*, and *TaMYB44* (Yang et al., 2012; Geng et al., 2017; Liu et al., 2023; He et al., 2025). Genes related to spike length include Q, *TaeEF1A*, and *TaAIRP2-1B* (Zhang et al., 2023; Liu et al., 2025; Yan et al., 2025). Additionally, genes such as *TaJAZ1*, *TaSPL13*, *GNI1*, and *WFZP* have been reported to be involved in the regulation of spike development (Sakuma et al., 2019; Li et al., 2021a; Gupta et al., 2023; Li et al., 2020; Jardon et al., 2023; Ye et al., 2024). Meanwhile, QTLs controlling TKW, GNS, spike length (SL), and spikelet number (SN) are widely distributed across all 21 chromosomes of wheat (Yang et al., 2021; Saini et al., 2022; Ding et al., 2024; Liu et al., 2024b). For example, *QSc/Sl.cib-5A* and *QSc/Sl.cib-6A* exhibit pleiotropic effects on spike length, spike density, and thousand-kernel weight (Li et al., 2021b). Despite considerable progress in research on yield potential, the underlying functional mechanisms and the inter-trait associations remain unclear.

With the development of high−density genotyping platforms such as 16K and 90K SNP chips, as well as high−throughput genotyping technologies like the kompetitive allele specific PCR (KASP), the capacity of genome−wide association studies (GWAS) and quantitative trait locus (QTL) mapping for dissecting the genetic basis of complex quantitative traits has been significantly enhanced (Muhammad et al., 2020; Malik et al., 2021; Chidzanga et al., 2022). Compared with traditional linkage analysis, GWAS has become a core method for mining key genetic loci associated with yield-related traits due to its higher mapping resolution and stronger adaptability to diverse populations (Breseghello and Sorrells, 2006). However, the effectiveness of GWAS highly depends on the genetic diversity of the population used (Andrijanić et al., 2023). In addition to modern cultivars, landraces harboring abundant genetic variations represent a valuable reservoir for discovering favorable alleles (Bailey-Serres et al., 2019; Liu et al., 2024a). Therefore, exploration of yield−related genetic loci in landraces is of great significance for advancing molecular breeding.

Based on this, we selected 372 wheat landraces mainly originating from Gansu, China. Four key yield-related traits: TKW, SL, GNS, and SN were measured in multi-environment field trials over multiple years and locations. Whole-genome genotyping was performed using a 16K liquid-phase SNP chip. By integrating phenotypic and genotypic data, we conducted a genome-wide association study to identify genetic loci significantly associated with the above-mentioned traits. The study aims to provide directly applicable genetic resources and molecular marker targets for wheat molecular breeding.

## 2 Materials and methods

### 2.1 Plant materials and phenotypic analysis

This study systematically phenotyped 372 wheat landrace accessions obtained from the Gansu Provincial Crop Germplasm Resources Bank. All materials were cultivated over four consecutive wheat growing seasons (2021-2022, 2022-2023, 2023-2024, and 2024-2025, abbreviated as 22GS, 23GS, 24GS, and 25GS, respectively) at the Qingshui Experimental Station of the Gansu Academy of Agricultural Sciences. A single wheat growing season (abbreviated as 24LZ) was planted at the Qinwangchuan Modern Agriculture Comprehensive Experimental Station of the Gansu Academy of Agricultural Sciences in 2023-2024. The field trial adopted a single-row planting design with a row length of 1.5 m and a row spacing of 0.3 m. Indoor evaluation recorded SL, SNS, GNS, and TKW measured by weighing. All phenotypic data were averaged across replicates to obtain the final phenotypic values under each growing environment.

Descriptive statistical analysis was performed on the phenotypic observations in each environment and on the multi−environment Best Linear Unbiased Estimates (BLUEs), including basic statistics such as minimum, maximum, mean, and coefficient of variation. The broad−sense heritability (*H²*) was estimated using the following formula: $H^{2}=\frac{\sigma_{G}^{2}}{\sigma_{G}^{2}+\frac{\sigma_{e}^{2}}{e}}$. where *σ_G²* is the genotypic variance component, *σ_e²* is the residual variance component that includes genotype-by-environment interaction and random error, and e is the number of environments. The standard error of heritability was estimated using the Delta method, and its 95% confidence interval was calculated via 1,000 parametric bootstrap replicates to evaluate the robustness of the estimation. All statistical analyses were performed in the R 4.3.2 environment, mainly utilizing the R packages lme4 (v1.1-35.1), emmeans (v1.8.9), and ggplot2 (v3.4.4) (Bates et al., 2015; Lenth, 2016; Wickham, 2016).

### 2.2 DNA extraction and genotyping

Leaf samples from 372 Gansu landrace accessions were sent to Molbreeding Biotechnology Co., Ltd. (https://www.molbreeding.com) in Shijiazhuang, Hebei Province for genotyping using the wheat 16K liquid-phase chip. The physical positions of the single nucleotide polymorphism (SNP) markers were anchored to the Chinese Spring reference genome sequence RefSeq v2.1 (Zhu et al., 2021). Quality control of the genotype data was performed using PLINK software (Purcell et al., 2007). SNP markers with a missing rate higher than 20% and a minor allele frequency lower than 5% were removed. The remaining high-quality polymorphic SNPs were retained for subsequent analysis.

### 2.3 Population structure and linkage disequilibrium

To elucidate the population genetic structure of the samples, analysis was performed using the ADMIXTURE software (version 1.3.0) (Chakraborty and Weiss, 1988). Multiple runs were conducted by setting the number of assumed ancestral populations (K) from 1 to 20. The optimal K value was determined by comparing the cross-validation (CV) errors corresponding to different K values. To investigate the decay characteristics of linkage disequilibrium (LD) within the genome, analyses were performed separately for the A, B, and D subgenomes as well as the complete ABD genome using the PopLDdecay software (Zhang et al., 2019). The maximum physical distance between marker pairs was set to 10,000 bp. The LD decay curves were visualized using the ggplot2 package in R. The LD decay distance was defined as the physical distance at which the linkage disequilibrium coefficient (*r²*) drops to half of its maximum value within a 10 Mb range. This decay distance was separately calculated for each subgenome and for the whole genome.

### 2.4 Genome-wide association study

This study performed GWAS for the traits SL, GNS, SNS, and TKW using the GAPIT3 software (Wang and Zhang, 2021). To account for the effects of population structure and kinship, the samples were divided into five subgroups, and the kinship matrix calculated by GAPIT was incorporated into the model as a random effect. A Mixed Linear Model (MLM) was employed for the primary analysis. Additionally, supplementary validation was conducted using multiple models: the Multi-Locus Mixed Model (MLMM), Fixed and Random Model Circulating Probability Unification (FarmCPU), Bayesian-information and Linkage-disequilibrium Iteratively Nested Keyway (BLINK), and Compressed Mixed Linear Model (CMLM). These models were used to analyze the association between each phenotype and genotype, thereby enhancing the reliability of identified loci.

To control false positives due to multiple testing while retaining the power to detect moderate-effect loci, we adopted a dual-threshold strategy for SNP significance. For each single-model single-environment analysis, we applied the Benjamini-Hochberg false discovery rate (FDR) correction to the raw P-values. A SNP was considered significant if it satisfied both of the following criteria: (i) raw *P* < 1×10^-4^ (suggestive threshold), and (ii) FDR-adjusted q < 0.10. The combination of a nominal threshold (*P* < 1×10^-4^) and an FDR control (*q* < 0.10) balances statistical stringency and discovery power, as commonly recommended for GWAS in plants (Benjaamini and Hochberg, 1995; Sadeqi et al., 2025). Importantly, to further minimize false positives arising from single-model or single-environment artifacts, a locus was considered reliable only if it was detected in at least two different GWAS models (e.g., MLM and FarmCPU) and in at least one single environment plus the multi-environment Best Linear Unbiased Estimates (BLUE) analysis, meeting the above significance criteria (raw *P* < 1×10^-4^ and FDR *q* < 0.10) in each of those tests. This multi-model, multi-environment consensus strategy serves as a stringent biological filter.

To evaluate the distribution of test statistics and their significance, quantile-quantile (Q-Q) plots and Manhattan plots were generated using the R package CMplot. Furthermore, to quantify the contribution of each significant SNP to phenotypic variation, the Phenotypic Variation Explained (PVE) was calculated according to the method described by Shim et al. The formula used was: $PVE=\frac{2\times Effect^{2}\times MAF\times (1-MAF)}{Var_{Y}}$, where MAF is the minor allele frequency and Effect refers to the estimated additive effect.

### 2.5 Haplotype analysis and candidate locus selection

Haplotype analysis was performed in R for the significant loci identified by GWAS. For each locus, genotypes of the lead SNP and its flanking SNPs within the LD decay distance were extracted from the 16K SNP dataset, and haplotypes were defined based on allele combinations across SNPs. To ensure statistical reliability, only haplotypes with a frequency of at least three accessions were retained for analysis.

Due to highly unbalanced sample sizes among haplotype groups and non-normally distributed phenotypic data, non−parametric tests were used for group comparisons. For loci with only two haplotypes, the Mann−Whitney U test (Wilcoxon rank−sum test) was applied; for loci with three or more haplotypes, the Kruskal−Wallis test was first performed, followed by Dunn’s post−hoc test with Bonferroni correction for multiple comparisons. All tests were conducted separately for each single environment and for the multi−environment BLUE values, with a significance threshold of *P* < 0.05.

Loci showing significant haplotype effects (P < 0.05) in both the BLUE analysis and at least two single environments were considered to have robust associations. Finally, candidate genes were predicted within the physical intervals of these loci based on the Chinese Spring reference genome (RefSeq v2.1) and their expression patterns in wheat tissues (Zhu et al., 2021).

### 2.6 Candidate genes prediction

Candidate genes were predicted for the newly identified loci that were consistently detected across multiple environments, and were not previously reported as known QTLs. For each significant locus, its physical interval was defined based on the LD decay distance of its chromosome. Genes within these genomic intervals were identified using the Interval Tool (http://202.194.139.32/tools/intervalTools.html) on the Wheat Omics 1.0 platform (http://202.194.139.32/jbrowse.html). Following the removal of low-confidence genes, the expression patterns of the retained high-confidence genes across various wheat tissues were analyzed via the Hexaploid Wheat Expression Database tool (http://202.194.139.32/expression/wheat.html) within the Gene Expression (http://202.194.139.32/expression/index.html) platform. Based on this analysis, candidate genes associated with key agronomic traits in wheat were subsequently selected.

## 3 Results

### 3.1 Analysis of phenotypic variation in yield-related traits

**Figure 1:**
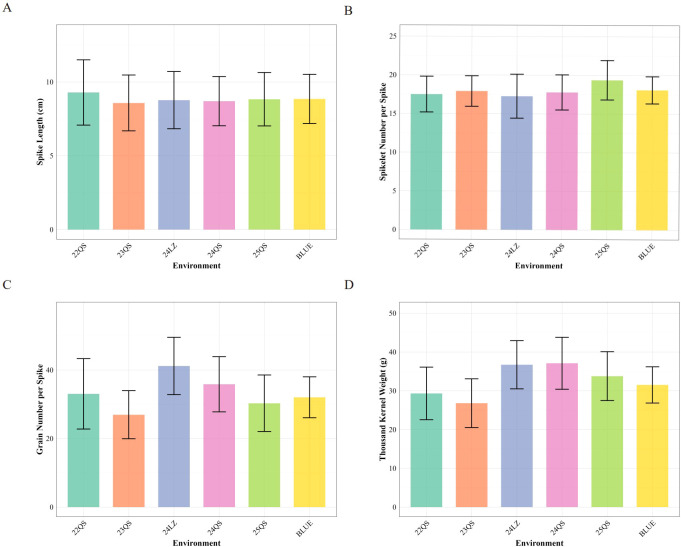
Phenotypic distributions of four agronomic traits in 372 Gansu wheat landraces across five environments. 24LZ (2024, Lanzhou, Gansu Province); 22QS (2022, Qingshui, Gansu Province); 23QS (2023, Qingshui, Gansu Province); 24QS (2024, Qingshui, Gansu Province); 25QS (2025, Qingshui, Gansu Province). BLUE, best linear unbiased estimate. **(A)** spike length (SL, cm); **(B)** spikelet number per spike (SNS); **(C)** grain number per spike (GNS); **(D)** thousand-kernel weight (TKW, g/1000 kernels).

Descriptive statistics for the four traits across five environments showed significant differences in their variation levels and genetic control (**Figure 1**; **Table 1**; **Supplementary Table 1**). SL were relatively consistent mean values across environments (approximately 8.58-9.29 cm), with low standard deviation and moderate relative variation (CV ≈ 19.16-23.80%). This trait exhibited the highest broad-sense heritability (*H²* ≈ 0.70), indicating strong genetic control and good environmental stability. In contrast, TKW varied considerably among environments (mean range 26.81–37.09 g), with a CV of 16.93%–23.44% and a lower heritability (*H²* ≈ 0.37), reflecting greater environmental influence. GNS displayed the largest phenotypic variation (mean range 26.98–41.20, CV up to 31.09%) and the lowest heritability (*H²* ≈ 0.35), highlighting its high environmental sensitivity and weak genetic control. SNS maintained relatively stable means across environments (mean range 17.28–19.37), with low CV (≈ 11.00-16.45%) and moderate heritability (*H²* ≈ 0.40). In summary, the traits differed markedly in their environmental response and genetic determinacy. SL was genetically stable, whereas TKW and GNS, despite showing wider phenotypic variation, were more susceptible to environmental modulation.

**Table 1:** Phenotypic variation and broad-sense heritability of thousand-kernel weight, spike length, grains per spike, and spikelet number in 372 Gansu wheat landraces across multiple environments.

Trait	Environment^a^	Max	Min	Mean	SD	CV^b^	*H* ^2c^
SL	24LZ	16.83	3.97	8.77	1.94	22.1	0.7
	22QS	17.67	4.5	9.29	2.21	23.8	
	23QS	16.67	3.73	8.58	1.89	22.04	
	24QS	14.43	4.33	8.7	1.67	19.16	
	25QS	14.5	3.5	8.83	1.81	20.48	
SNS	24LZ	25.33	10.33	17.28	2.84	16.45	0.4
	22QS	22.67	11.5	17.51	2.31	13.21	
	23QS	23.33	13	17.93	1.97	11	
	24QS	24.67	12.67	17.79	2.27	12.77	
	25QS	25.67	12	19.37	2.55	13.15	
GNS	24LZ	60.4	20	41.2	8.37	20.3	0.35
	22QS	59.52	15.18	33.08	10.28	31.09	
	23QS	51.5	15	26.98	7.02	26.03	
	24QS	61.56	18	35.86	8.05	22.46	
	25QS	62.35	15.2	30.32	8.25	27.22	
TKW	24LZ	49.97	19.21	36.71	6.21	16.93	0.37
	22QS	47.2	15.36	29.33	6.77	23.1	
	23QS	46.58	15.04	26.81	6.28	23.44	
	24QS	49.92	21.29	37.09	6.69	18.05	
	25QS	50.82	20.49	33.78	6.3	18.66	

a 24LZ (2024, Lanzhou, Gansu Province); 22QS (2022, Qingshui, Gansu Province); 23QS (2023, Qingshui, Gansu Province); 24QS (2024, Qingshui, Gansu Province); 25QS (2025, Qingshui, Gansu Province).

b CV, coefficient of variation.

c *H*^2^, broad-sense.

### 3.2 Correlations among yield-related traits

In the phenotypic correlation analysis, correlation coefficients for the same trait across different years or locations were predominantly positive and highly significant, indicating strong cross-environment repeatability in genetic expression. For example, SL exhibited the highest inter-annual correlation coefficients (0.61-0.79) and correlated strongly with its BLUE values, suggesting that SL is the least environmentally sensitive and the most stable. TKW showed moderate consistency across environments (0.23-0.71), and its BLUE values were significantly correlated with performance in most individual environments, supporting the utility of BLUE values for integrating multi-year data and reflecting population-level genetic potential.

Although inter-annual correlations for SNS and GNS were positive, they display were relatively more variable (0.18-0.56). Most correlation pairs had extremely low p-values, indicating that BLUE values also effectively integrate grain-number traits, despite their higher susceptibility to environmental regulation compared to SL and TKW.

**Figure 2:**
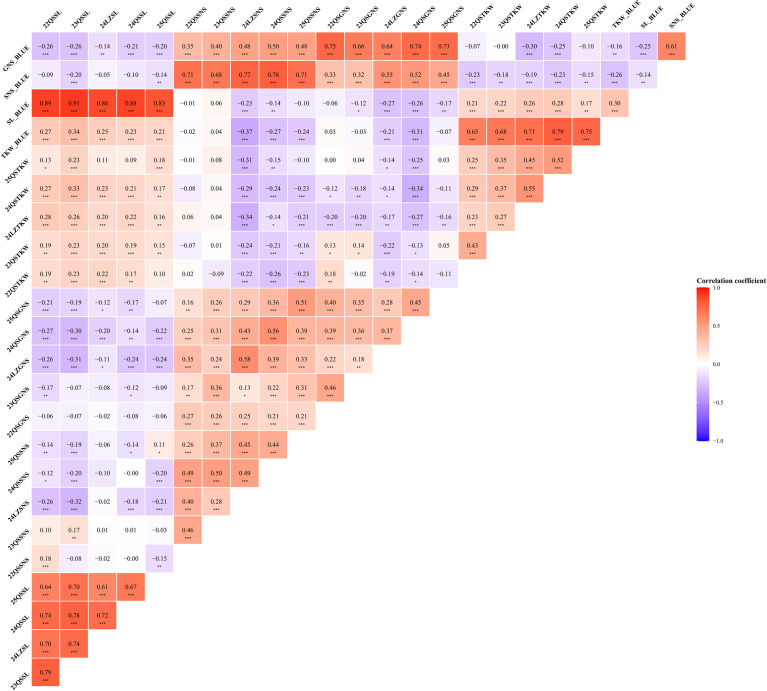
Heatmap of correlation coefficients of agronomic traits for 372 Gansu wheat landraces under six environmental conditions. Colors ranging from blue (negative correlation) to red (positive correlation) indicate the magnitude of the correlation coefficient; *P<0.05, **P<0.01, ***P<0.001.

Regarding trait-trait genetic associations, SNS and GNS showed significant positive correlations in most environments, suggesting that higher spikelet numbers generally lead to more grain number per spike. Conversely, GNS and TKW exhibited significant negative correlations in some environments, supporting a resource-allocation trade-off between grain number and grain size. SL and TKW generally displayed weak to moderate positive correlations, most of which were statistically significant (*r^2^* ≈ 0.10-0.34) (**Figure 2**).

### 3.3 SNP marker distribution, population structure, and linkage disequilibrium analysis

After quality control filtering (removing sites with a missing rate > 20% and a minor allele frequency < 5%), a total of 13,218 high-quality polymorphic SNP markers were retained, covering all 21 chromosomes. marker distribution across chromosomes was uneven. The A, B, and D genomes harbored 6,305, 6,271, and 642 markers, accounting for approximately 47.7%, 47.4%, and 4.9% of the total markers, respectively, with the D genome showing a notably lower abundance. The disparity was even more pronounced at the chromosomal level: chromosome 3B carried the highest number of markers (1,200, constituting 9.08% of the total), whereas chromosome 5D had the fewest, accounting for only 0.35%. The markers spanned a total genomic length of 14,066.28 Mb, corresponding to an average marker density of 0.94 SNP/Mb.

**Figure 3:**
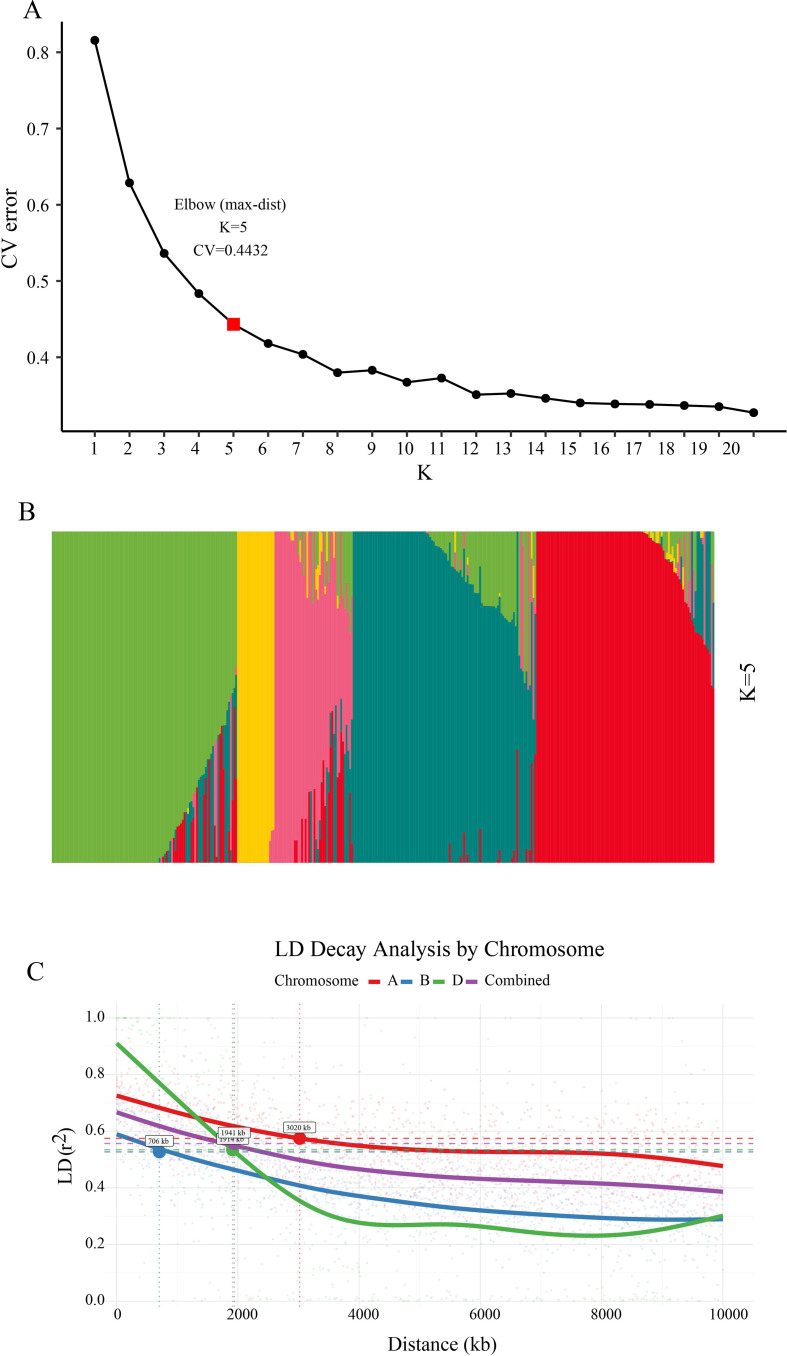
Analysis of population structure of 372 Gansu wheat landraces. **(A)** The distribution of CV error values from ADMIXTURE analysis. **(B)** Population structure of 372 accessions of the wheat landraces natural population. **(C)** Linkage disequilibrium (LD)decay across the whole genome and the **(A, B, D)** sub-genomes.

Population structure analysis using Admixture revealed a distinct inflection point in the cross-validation error rate at *K* = 5 (**Figure 3A**), indicating that the panel of accessions could be optimally divided into five subpopulations (**Figure 3B**). Linkage LD decay analysis showed that the LD decay distances for the A, B, and D genomes were 3.02 Mb, 0.71 Mb, and 1.91 Mb, respectively, with a genome-wide average of 1.94 Mb (**Figure 3C**). The B genome exhibited the fastest LD decay rate, suggesting that this subgenome may have experienced stronger selection pressure in the panel of Gansu local wheat varieties.

### 3.4 Loci associated with yield-related traits identified by GWAS

**Figure 4:**
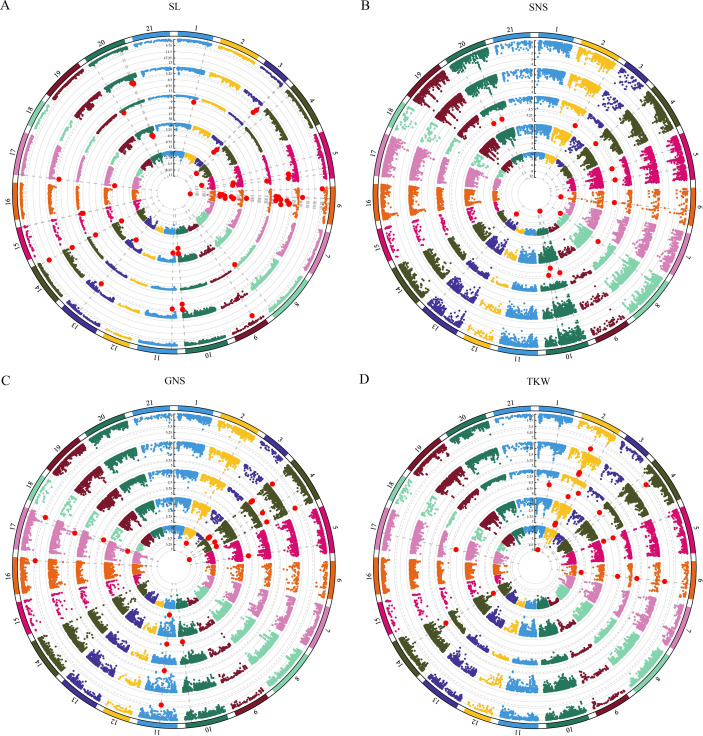
Manhattan plots for BLUE under different yield-related traits. Images **(A-D)** represent Manhattan plots generated from genome-wide association studies (GWAS) for BLUE under different traits (SL, SNS, GNS, TKW). The Manhattan plots from the center outward correspond to different models in sequence: BLINK, CMLM, FarmCPU, MLM, MLMM.

**Figure 5:**
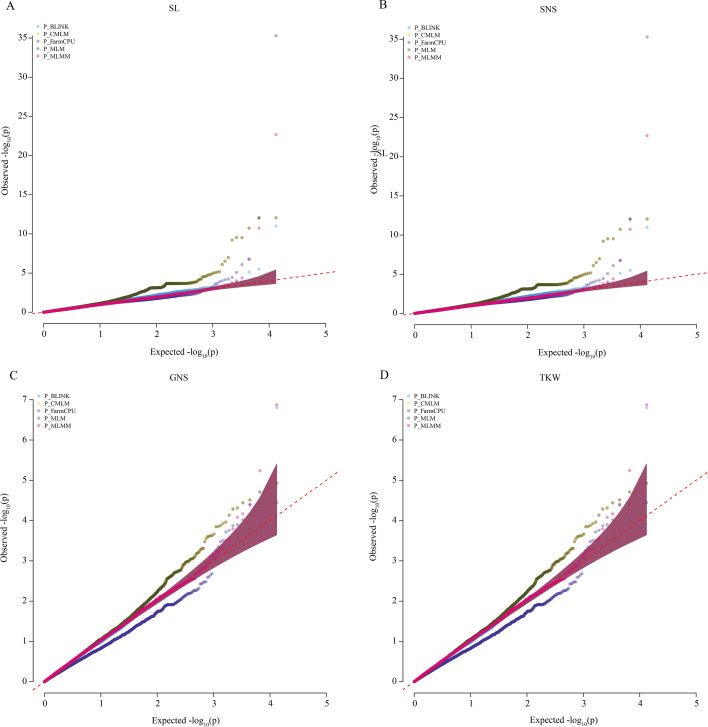
Q-Q plots for BLUE under different yield-related traits. Images **(A-D)** represent Quantile-quantile (Q-Q) plots generated from genome-wide association studies (GWAS) for the BLUE under different traits (SL, SNS, GNS, TKW).

A total of 19 significant genetic loci associated with four yield-related traits: SL, GNS, SNS, and TKW (**Table 2**; **Figures 4**, **5**; **Supplementary Figures 1**–**4**) have been mapped by GWAS. Among these, nine loci were associated with SL, including *QSl.gaas-2D.1* (PVE = 18%), *QSl.gaas-2D.2* (20%), *QSl.gaas-3B* (5%), *QSl.gaas-4A* (11%), *QSl.gaas-4B* (17%), *QSl.gaas-5A* (4%), *QSl.gaas-5B.1* (6%), *QSl.gaas-5B.2* (22%), and *QSl.gaas-7B* (9%), with *QSl.gaas-5B.2* exhibiting the highest contribution rate. Four loci were associated with GNS: *QGns.gaas-2A.1* (5%), *QGns.gaas-2A.2* (11%), *QGns.gaas-2A.3* (5%), and *QGns.gaas-7A* (7%). One loci were identified for SNS: *QSns.gaas-7A* (6%). For TKW, five loci were detected: *QTkw.gaas-1B* (9%), *QTkw.gaas-2B* (20%), *QTkw.gaas-2D* (6%), *QTkw.gaas-4B* (5%), *QTkw.gaas-5B* (2%). These findings provide critical insights into genetic basis of yield-related traits in wheat and offer valuable resources for subsequent molecular marker-assisted breeding.

**Table 2:** Identification of 19 significant loci for wheat spike length, spikelet number per spike, grain number per spike, and thousand−kernel weight under multi-environment and multi-model conditions.

Genetic loci	Trait	Peak marker	Chr	Physical intervala	PVE	SNP of num.	Other model	Environment	Reported locus	Other yield-related trait loci	Reference
*QSl.gaas-2D.1*	SL	*2D_201737062*	2D	203894528-213038784	18%	2	BLINK, CMLM, FarmCPU, MLMM	22QS, 23QS, 24QS, 24LZ, 25QS, BLUE			
*QSl.gaas-2D.2*	SL	*2D_313801190*	2D	316345532	20%	1	BLINK, CMLM, FarmCPU, MLMM	22QS, 23QS, 24QS, 24LZ, 25QS, BLUE			
*QSl.gaas-3B*	SL	*3B_586543446*	3B	601632954	5%	1	BLINK, CMLM, FarmCPU, MLMM	24LZ, BLUE			
*QSl.gaas-4A*	SL	*4A_720066506*	4A	726759366-728341124	11%	3	CMLM, FarmCPU	22QS, 23QS, 24QS, 24LZ, 25QS, BLUE	Qsl.nhv-4A.a (715.30–722.41 Mb)		Chandra et al., 2025
									*MQTL-4A.3* (728.44-742.07 Mb)		Yang et al., 2024
*QSl.gaas-4B*	SL	*4B_94321322*	4BS	97320125	17%	1	CMLM, MLMM	22QS, 23QS, 24QS, 24LZ, BLUE		*QKnps.hwwgr-4BS* (78.78-101.70 Mb)	Li et al., 2015
*QSl.gaas-5A*	SL	*5A_552747815*	5A	553400727	4%	1	CMLM, MLMM	22QS, BLUE	*QSL.sxau-5A* (451.87-595.42-681.45 Mb)	NSPS (552.5 Mb)	Qiao et al., 2022; Halder et al., 2023
									*QSl.cau-5A* (520.63-547.11 Mb)	QKns/Tss/Fss/SC.haust-5A (547.49-590.46 Mb)	Zhai et al., 2016; Wang et al., 2023
									*QSl.cas-5A.2* (537.36-539.09 Mb)	*QSc.sxau-5A* (451.87-595.42-681.45 Mb)	Liu et al., 2022; Qiao et al., 2022
									*QSl.cas-5A.3* (598.02-598.53 Mb)	*QKns.his-5A-2* (549.11-574.74 Mb)	Liu et al., 2022; Xu et al., 2022
										*QSc.cau-5A* (520.63-547.11 Mb)	Zhai et al., 2016
*QSl.gaas-5B.1*	SL	*5B_398349721*	5B	401346343	6%	1	CMLM, MLMM	24QS, BLUE			
*QSl.gaas-5B.2*	SL	*5B_674738376*	5B	675559215	22%	1	BLINK, CMLM, FarmCPU	22QS, 23QS, 24QS, 24LZ, BLUE	*QSl.yaas-5B* (642.52-658.83 Mb)		Zhao et al., 2023
*QSl.gaas-7B*	SL	*7B_619667518*	7B	625168206-626086743	9%	3	CMLM, MLMM	23QS, 24QS, 24LZ, BLUE			
*QGns.gaas-2A.1*	GNS	*2A_82137771*	2AS	86806096-86806143	5%	3	BLINK, CMLM, FarmCPU,MLMM	22QS, 23QS, BLUE	Qgns.iiwbr-2A (85.55 Mb)	*qSL-2A.2* (82.64 Mb)	Bhusal et al., 2017; Liu et al., 2018
*QGns.gaas-2A.2*	GNS	*2A_487685973*	2A	486914786-492301204	11%	3	CMLM, MLMM	23QS, BLUE			
*QGns.gaas-2A.3*	GNS	*2A_744903829*	2A	748913954	5%	1	BLINK, CMLM, FarmCPU, MLMM	24QS, BLUE	*QKns.mgb-2A* (727.27-748.48 Mb)	QSL.caas-2AL (739.25-740.36 Mb)	Mangini et al., 2018; Gao et al., 2015
										QSC.cib-CK1-2A.2 (744.95-748.66 Mb)	Li et al., 2021a
*QGns.gaas-7A*	GNS	*7A_623435862*	7A	626753438-627438929	7%	2	CMLM, MLMM	23QS, BLUE	QKps.mgb-7A.1 (538.93-629.74 Mb)	SL (610.9 Mb)	Mangini et al., 2018; Halder et al., 2023
*QSns.gaas-7A*	SNS	*7A_86112295*	7AS	89735424	6%	1	CMLM, FarmCPU, MLMM	24LZ, BLUE	QSpn.nau-7A (68.7-93.7 Mb)		Ma et al., 2007
*QTkw.gaas-1B*	TKW	*1B_373003169*	1B	375671084-386491650	9%	8	BLINK, CMLM, FarmCPU.MLMM	22QS, BLUE	TaPYL1-1B (373.62 Mb)		Mao et al., 2021
*QTkw.gaas-2B*	TKW	*2B_71315735*	2BS	75511921 -79703329	20%	10	BLINK, CMLM, FarmCPU,MLMM	25QS, BLUE	*QTkw.macs-2B* (81.16 Mb)	QKws.macs-2B (81.16 Mb)	Patil et al., 2013
*QTkw.gaas-2D*	TKW	*2D_391463774*	2D	393700939	6%	1	BLINK, CMLM, FarmCPU,MLMM	23QS, BLUE		qSl-2D (390.84-480.44 Mb)	Fan et al., 2019
*QTkw.gaas-4B*	TKW	*4B_101250000*	4BS	104140731-109880306	5%	5	FarmCPU, MLMM	22QS, 24LZ, BLUE		QKnps.hwwgr-4BS (78.78-101.70 Mb)	Li et al., 2015
*QTkw.gaas-5B*	TKW	*5B_504301648*	5B	507262600	2%	1	BLINK, FarmCPU, MLMM	24QS, BLUE	*QTgw-5B* (509.55-537.85 Mb)	SL (513.9 Mb)	Roncallo et al., 2017; Halder et al., 2023
									*QTkw.mgb-5B* (475.36-510.42 Mb)	SL, *MQTL-5B.2* (502.02-511.59 Mb)	Mangini et al., 2018; Yang et al., 2024
									*TaPRR95* (505.80 - 505.81 Mb)		Fu et al., 2024

a The physical positions were determined based on the Chinese Spring reference genome IWGSC RefSeq v2.1.

### 3.5 Haplotype analysis of the landrace

**Figure 6:**
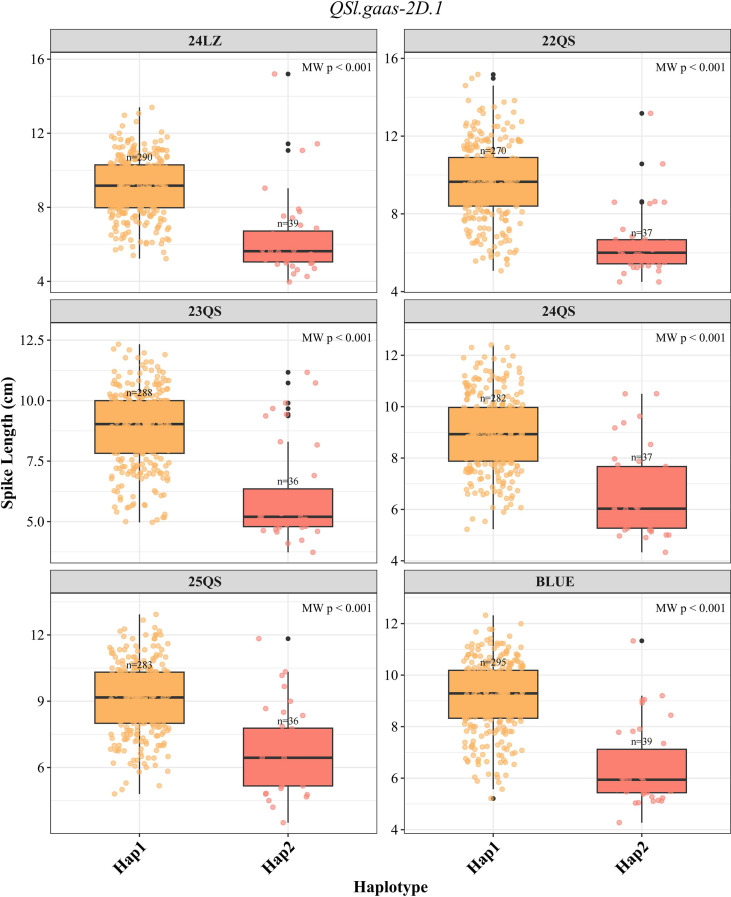
Haplotype analysis of SL loci *QSl.gaas-2D.1.*Box plots showing spike length differences between Hap1 and Hap2 in six environmental conditions (24LZ, 22QS, 23QS, 24QS, 25QS, BLUE). MW, Mann−Whitney U test; *p*, p−value; n, Sample size; The same as **Figures 7**, **8**.

**Figure 7:**
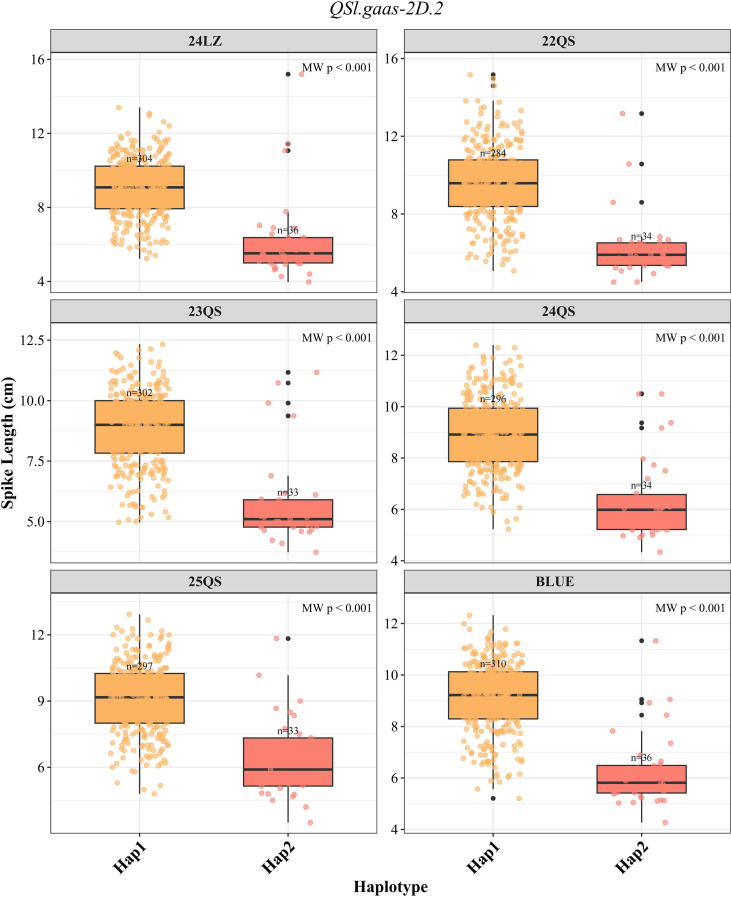
Haplotype analysis of SL loci *QSl.gaas-2D.2*.

**Figure 8:**
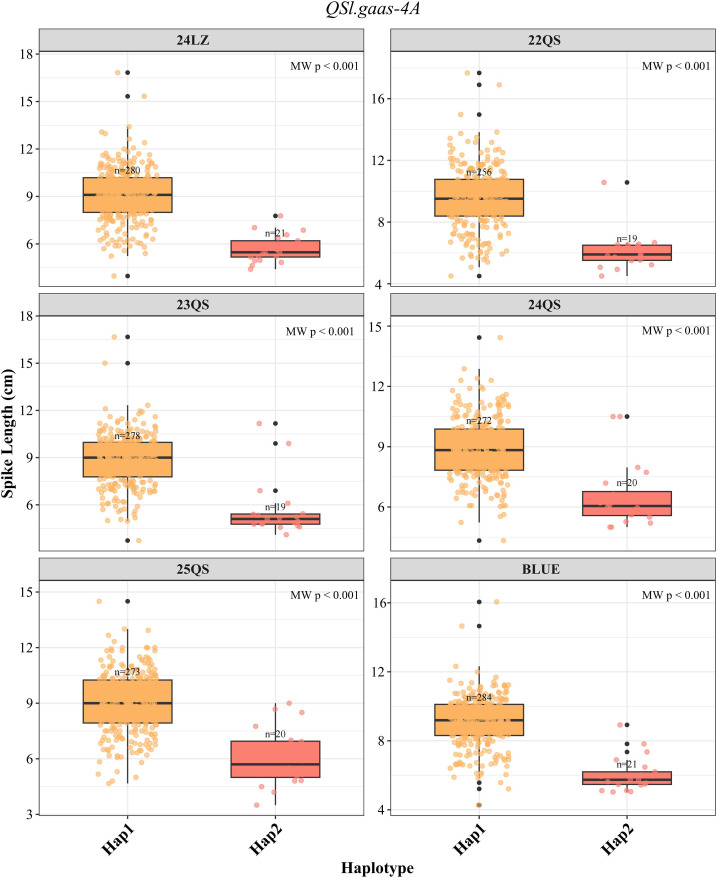
Haplotype analysis of SL loci *QSl.gaas-4A*.

To investigate the association between the newly identified loci and yield-related traits, we performed haplotype analysis on 19 previously mapped loci. The SL locus *QSl.gaas-2D.1* exhibited two major haplotypes (**Figure 6**): Hap1 (G-A, n=295) had a mean SL of 9.16 cm, significantly higher than Hap2 (A-G, n=39, 6.45 cm). Similarly, *QSl.gaas-2D.2* also presented two major haplotypes (**Figure 7**): Hap1 (G, n=310) showed a mean SL of 9.13 cm, significantly exceeding that of Hap2 (A, n=36, 6.21 cm). For *QSl.gaas-3B*, three haplotypes were identified. No significant difference in SL was observed between Hap1 (G-T, n=256, 9.15 cm) and Hap2 (G-C, n=16, 8.48 cm), whereas Hap3 (A-C, n=28, 7.19 cm) had a significantly lower mean SL, suggesting it may represent a deleterious haplotype (**Supplementary Figure 6A**). *QSl.gaas-4A* contained two major haplotypes (**Figure 8**): Hap1 (G-C-T, n=284) had a mean SL of 9.12 cm, significantly higher than that of Hap2 (C-T-C, n=21, 6.04 cm). *QSl.gaas-4B* also comprised two major haplotypes (**Supplementary Figure 6B**): Hap1 (G, n=351) and Hap2 (A, n=17) exhibited mean SL of 8.86 cm and 8.84 cm, respectively, with no significant difference. Similarly, *QSl.gaas-5A* presented two major haplotypes (**Supplementary Figure 6C**): Hap1 (A, n=222) and Hap2 (A, n=74) had mean SL of 8.81 cm and 8.79 cm, respectively, and the difference was not statistically significant. For *QSl.gaas-5B.1*, two major haplotypes were detected (**Supplementary Figure 6D**): Hap1 (C, n=325) showed a mean SL of 9.02 cm, significantly higher than Hap2 (A, n=31, 7.44 cm). *QSl.gaas-5B.2* also had two major haplotypes (**Supplementary Figure 6E**): Hap1 (G, n=353) and Hap2 (A, n=18), exhibited mean SL of 8.85 cm and 8.81 cm, respectively, with no significant difference. Finally, *QSl.gaas-7B* exhibited two major haplotypes (**Supplementary Figure 6F**): Hap1 (T-A-A, n=345) and Hap2 (G-C-G, n=21) had mean SL of 8.85 cm and 8.62 cm, respectively, with no significant difference between them.

The SNS-associated loci *QSns.gaas-7A* presents two major haplotypes (**Supplementary Figure 7**): Hap1 (A, n=178) and Hap2 (C, n=129) showed mean SNS of 18.27 and 17.91, respectively, with no significant difference between them.

**Figure 9:**
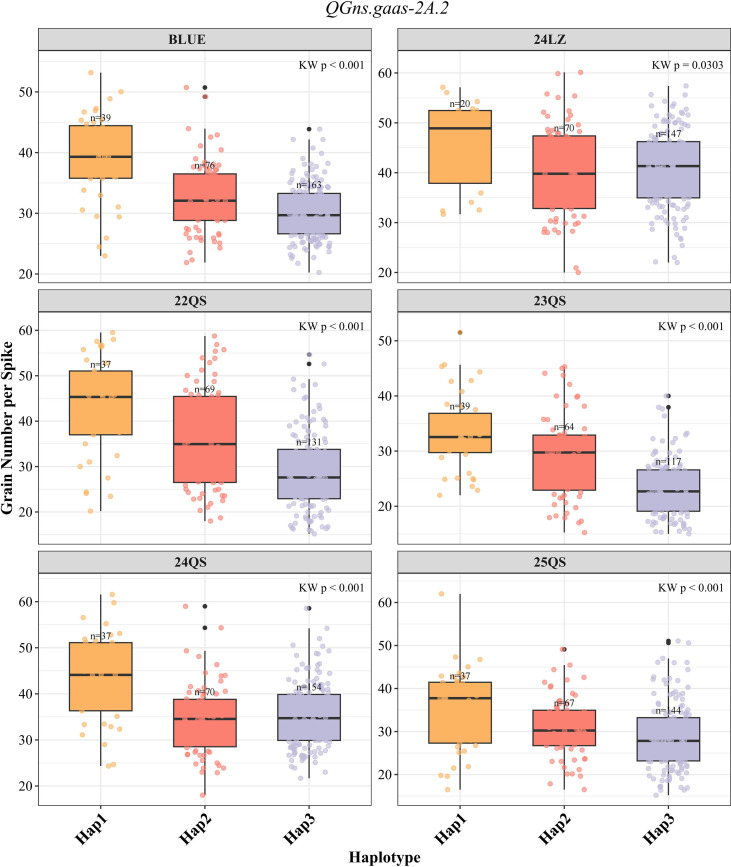
Haplotype analysis of GNS loci *QGns.gaas-2A.2.*Box plots showing grain number per spike among three haplotypes (Hap1, Hap2, Hap3) in six environmental conditions (24LZ, 22QS, 23QS, 24QS, 25QS, BLUE). KW, Kruskal-Wallis test; *p*, p−value; n, Sample size. The same as **Figures 10**.

For GNS, the locus *QGns.gaas-2A.1* exhibits three haplotypes (**Supplementary Figure 8A**): Hap1 (C-A-C, n=52) had the highest average grain number (38.99 grains), significantly exceeding that of Hap2 (C-G-C, n=152, 31.01 grains) and Hap3 (G-G-T, n=90, 30.66 grains). *QGns.gaas-2A.2* also comprised three haplotypes (**Figure 9**): Hap1 (T-A-A, n=39) had the highest mean grain number (39.11 grains), significantly higher than Hap2 (C-G-G, n=76, 32.62 grains) and Hap3 (T-G-A, n=163, 30.18 grains). *QGns.gaas-2A.3* carried two haplotypes (**Supplementary Figure 8B**), Hap1 (T, n=30) produced an average of 38.03 grains, significantly higher than Hap2 (C, n=316, 31.44 grains). *QGns.gaas-7A* possessed two haplotypes (**Supplementary Figure 8C**): Hap1 (C-T, n=22) had an average grain number of 37.18 grains, significantly higher than that of Hap2 (T-A, n=341, 31.60 grains).

**Figure 10:**
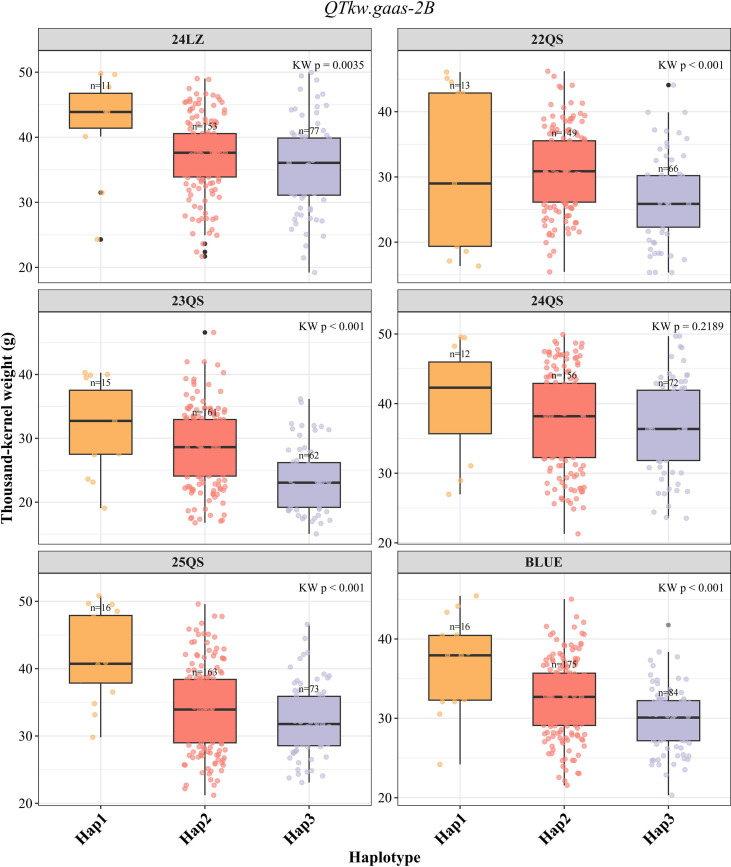
Haplotype analysis of TKW loci *QTkw.gaas-2B*.

For TKW, the locus Q*Tkw.gaas-1B* comprise two major haplotypes (**Supplementary Figure 9A**): Hap1 (C-G-A-C-T-T-G-C, n=30) and Hap2 (T-C-G-T-C-C-C-T, n=300) showed mean values of 32.20 g and 31.59 g, with no significant difference. *QTkw.gaas-2B* exhibited three haplotypes (**Figure 10**): Hap1 (C-G-T-A-T-A-A-G-A-C, n=16) had the highest mean TKW of 36.83 g, followed by Hap2 (C-G-C-G-C-G-G-A-C-T, n=175, 32.52 g) and Hap3 (C-G-C-G-C-G-G-A-C-T, n=84, 29.84 g), with significant differences among all pairwise comparisons. *QTkw.gaas-2D* presented two haplotypes (**Supplementary Figure 9B**): Hap1 (A, n=53) exhibited a significantly higher mean TKW (34.58 g) than Hap2 (G, n=243, 30.20 g), indicating that the A allele is favorable. *QTkw.gaas-4B* harbored four haplotypes (**Supplementary Figure 9C**), Hap1 (G-G-G-G-C, n=13) had the highest TKW (36.53 g), followed by Hap2 (A-A-A-G-C, n=7, 34.65 g), Hap3 (G-G-G-G-T, n=12, 31.80 g), and Hap4 (A-A-A-A-T, n=294, 31.10 g), with a significant difference between Hap1 and Hap4. *QTkw.gaas-5B* had two haplotypes (**Supplementary Figure 9D**): Hap1 (A, n=41) showed a significantly higher mean TKW (35.83 g) than Hap2 (G, n=295, 30.88 g).

### 3.6 Prediction of candidate genes

We employed the Chinese Spring reference genome v2.1 to delineate the loci intervals based on chromosome-specific linkage disequilibrium decay distance. These intervals were subsequently used for gene annotation and candidate gene mining. The selected loci included *QSl.gaas-2D.1*, *QSl.gaas-2D.2*, *QGns.gaas-2A.2* and *QTkw.gaas-4B* (**Table 3**).

**Table 3:** Predicted genes within the candidate intervals of *QSl.gaas-2D.1, QSl.gaas-2D.2, QGns.gaas-2A.2* and *QTkw.gaas-4B*.

Trait	Loci	Chrom	Start	END	Gene03G	Strand	Function description
SL	*QSl.gaas-2D.1*	chr2D	203.253113Mb	203.256041Mb	*TraesCS2D03G0487500*	Forward	Ankyrin repeat domain protein, putative
		chr2D	206.647744Mb	206.653484Mb	*TraesCS2D03G0492100*	Forward	Serine/threonine protein phosphatase 7 long form isogeny
		chr2D	208.787966Mb	208.791965Mb	*TraesCS2D03G0494300*	Forward	Squamosa promoter-binding protein
	*QSl.gaas-2D.2*	chr2D	316.863166Mb	316.864991Mb	*TraesCS2D03G0596700*	Reverse	F-box family protein
		chr2D	316.885829Mb	316.88728Mb	*TraesCS2D03G0596800*	Reverse	B3 domain protein (DUF313)
GNS	*QGns.gaas-2A.2*	chr2A	492.74912Mb	492.749876Mb	*TraesCS2A03G0717100*	Reverse	ferredoxin-NADP -oxidoreductase 2
		chr2A	493.620822Mb	493.624063Mb	*TraesCS2A03G0717300*	Forward	Carboxypeptidase
TKW	*QTkw.gaas-4B*	chr4B	105.445347Mb	105.447171Mb	*TraesCS4B03G0223800*	Reverse	Heavy metal-associated protein
		chr4B	105.968528Mb	105.970747Mb	*TraesCS4B03G0224200*	Forward	Aspartic proteinase Asp1
		chr4B	106.510332Mb	106.513428Mb	*TraesCS4B03G0224600*	Forward	carboxyl-terminal peptidase (DUF239)
		chr4B	106.845752Mb	106.846384Mb	*TraesCS4B03G0225200*	Reverse	Pre-mRNA splicing factor, putative
		chr4B	106.960307Mb	106.960717Mb	*TraesCS4B03G0225300*	Reverse	Pre-mRNA splicing factor, putative
		chr4B	107.29636Mb	107.296992Mb	*TraesCS4B03G0226000*	Forward	Pre-mRNA splicing factor, putative
		chr4B	107.692391Mb	107.692852Mb	*TraesCS4B03G0226400*	Reverse	RING/U-box superfamily protein
		chr4B	108.434288Mb	108.435578Mb	*TraesCS4B03G0227100*	Reverse	Sphingoid base hydroxylase 2
		chr4B	109.145986Mb	109.148206Mb	*TraesCS4B03G0228800*	Reverse	F-box family protein

Heatmap analysis of candidate gene expression for spike length in different tissues (**Table 3**; **Supplementary Figure 5A**) revealed that *TraesCS2D03G0494300*, *TraesCS2D03G0487500*, *TraesCS2D03G0492100*, *TraesCS2D03G0596800*, and *TraesCS2D03G0596700* were highly expressed in wheat spikes, suggesting their potential role as candidate genes for spike length. These genes encode a Squamosa promoter-binding protein, a putative Ankyrin repeat domain protein; a Serine/threonine protein phosphatase 7 long form isogeny; and a B3 domain protein (DUF313), respectively.

Within the *QGns.gaas-2A.2* candidate interval, two genes *TraesCS2A03G0717100* and *TraesCS2A03G0717300* were found to be highly expressed in the wheat spikes (**Table 3**; **Supplementary Figure 5B**): These genes encode ferredoxin-NADP-oxidoreductase 2 and carboxypeptidase, respectively. In the TKW candidate interval, nine genes exhibiting high expression in wheat grains were identified from the expression heatmap (**Table 3**; **Supplementary Figure 5C**). These genes predominantly encode proteins such as heavy metal-associated protein, aspartic proteinase Asp1, carboxyl-terminal peptidase (DUF239), a putative Pre-mRNA splicing factor, RING/U-box superfamily protein, sphingoid base hydroxylase 2, and F-box family protein.

## 4 Discussion

### 4.1 Landraces in breeding and gene identification

Landraces harbor relatively unique and superior alleles, making them invaluable genetic resources for improving disease resistance, stress tolerance, and agronomic traits in crop breeding. Agronomic traits differ markedly between landraces and improved varieties. Landraces generally exhibit high genetic diversity, strong stress resistance, and distinctive quality characteristics, though their yield stability tends to be lower than that of improved varieties. For instance, Landraces typically have a lower TKW compared to improved varieties, attributed to their greater number of small spikelets, shorter spike length, and higher spikelet density. The traits such as compact spikes and multi-flowered spikes are key targets traits for variety improvement.

Landraces such as ‘Mazhamai’, ‘Zaoyangmai’, ‘Xibeifengshoumai’, and ‘Jiangdongmen’ have been successfully used as parents to develop improved varieties, including Bima 4, Beijing 8, Fengchan 3, Jinan 2, and Yangmai 5 (Zhuang, 2003). These varieties were widely cultivated from the 1950s to 1970s and made significant contributions to China’s food security. Through approaches such as exon sequencing, transcriptome sequencing, genome-wide association analysis, and gene cloning, numerous disease resistance genes and QTLs have been identified from wheat landraces. A notable example is *Yr18/Lr34/Pm38/Sr57*, is a well-characterized broad-spectrum and durable resistance gene derived from the Brazilian landrace ‘Frontana’ (Krattinger et al., 2009). The Fusarium head blight resistance gene *Fhb1* was identified from the Chinese landrace ‘Wangshuibai’ (Su et al., 2019). ‘WudouBaijianer’, a local wheat variety from Gansu, China, carries a dominant gene *YrWd*, effective against the CYR32 race and mapped to chromosome 4AL. It also harbors a recessive gene *yrWUD*, conferring resistance to V31/lab race, located within the 610.26-623.35 Mb region of chromosome 4AL based on the Chinese Spring reference genome v2.1 (Dong et al., 2024). Additionally, two stripe rust resistance genes, *YrBai* and *YrBDT*, were identified in the landrace ‘Baidatou’, conferring resistance to races CYR33 and CYR31, respectively (Wu et al., 2024). By utilizing these resistant landraces, such as those with white caterpillars and white large heads, a series of disease-resistant varieties have been developed, playing a pivotal role in the sustained management of wheat stripe rust in the hotspot Longnan of China.

### 4.2 Comparison of the major QTLs with previous results

In this study, a genome-wide association analysis was conducted using the wheat 16K gene chip to investigate SL, SN, GN, and TKW in 372 wheat landraces across multiple environments. A total of nine loci associated with spike length were identified, three of which overlapped with or were located close to previously reported QTLs. *QSl.gaas-4A* (726.59-728.34 Mb) was situated 4.18 Mb from the spike length *Qsl.nhv-4A.a* (715.30-722.41 Mb) (Chandra et al., 2025). *QSl.gaas-5A* (553.40 Mb) fell within the interval of *QSL.sxau-5A* (451.87-681.45 Mb) (Qiao et al., 2022) and was also near several other spike length QTLs, including *QSl.cau-5A* (520.63-547.11 Mb) (Zhai et al., 2016), *QSl.cas-5A.2* (537.36-539.09 Mb), and *QSl.cas-5A.3* (598.02-598.53 Mb) (Liu et al., 2022). *QSl.gaas-5B.2* (675.56 Mb) was located 16.73 Mb from *QSl.yaas-5B* (642.52-658.83 Mb) (Zhao et al., 2023). No previously reported spike length genes or QTLs were identified at the remaining six loci, suggesting they may represent novel loci.

For yield-related trait GNS, four loci were identified in these landraces. The previously reported *Qgns.iiwbr-2A* (85.55 Mb on chromosome 2AS; Bhusal et al., 2017) was approximately 1.26 Mb from the spikelet number locus *QGns.gaas-2A.1* (86.80 Mb). *QGns.gaas-2A.3* (748.91 Mb) was 0.43 Mb from the reported *QKns.mgb-2A* (727.27-748.48 Mb; Mangini et al., 2018). *QGns.gaas-7A* (626.75-627.44 Mb) was located 2.30 Mb from *QKps.mgb-7A.1* (538.93-629.74 Mb; Mangini et al., 2018). No known grain number-related genes or QTLs were identified within the interval of *QGns.gaas-2A.2*, indicating it may be a novel locus.

Regarding spikelet number per spike, *QSns.gaas-7A* (89.74 Mb) overlapped with the previously reported QTL *QSpn.nau-7A* (68.7-93.7 Mb; Ma et al., 2007).

A total of five TKW QTLs were identified, four of which overlapped with or were located near previously reported intervals. The wheat ABA receptor gene *TaPYL1-1B* (373.62 Mb on chromosome 1B, Mao et al., 2021), which enhances drought resistance and grain yield through improved water-use efficiency, was located approximately 0.62 Mb from *QTkw.gaas-1B*. *QTkw.gaas-2B* (75.51-79.70 Mb) was 1.46 Mb from the reported *QTkw.macs-2B* (81.16 Mb; Patil et al., 2013). The *TaPRR95* gene, which reduced plant height and increased thousand-grain weight, resides at 505.80 Mb on chromosome 5B (Fu et al., 2024) and is approximately 1.46 Mb from *QTkw.gaas-5B* (507.26 Mb).Two additional TKW QTLs; *QTgw-5B* (509.55-537.85 Mb; Roncallo et al., 2017) and *QTkw.mgb-5B* (475.36-510.42 Mb; Mangini et al., 2018) were detected near *QTkw.gaas-5B*. No previously reported TKW genes or QTLs were identified within the intervals of *QTkw.gaas-2D* and *QTkw.gaas-4B*, suggesting they may represent novel loci.

The coincidence of several loci with those identified in earlier studies reflects the structural complexity and linkage relationships within the wheat genome, while also reinforcing the reliability of the association mapping results presented here.

### 4.3 QTL cluster associated with yield-related traits

Pleiotropic or multifunctional loci are widely distributed in plant genomes and can influence multiple traits simultaneously. Among the loci detected in this study, several were found to colocalize with previously reported QTLs for other yield-related traits (**Table 3**). For instance, near the GNS locus *QGns.gaas-2A.3*, a previously reported SL QTL (*QSL.caas-2AL*) and a spikelet compaction QTL (*QSC.cib-CK1-2A.2*) have been documented (Gao et al., 2015; Li et al., 2021a). Similarly, two SL QTLs are located near the TKW locus *QTkw.gaas-5B* (Halder et al., 2023; Yang et al., 2024).

Notably, the *QSl.gaas-5A* locus on chromosome 5A resides within a region harboring at least five previously identified QTLs associated with diverse yield-related traits, including SN, spikelet compaction, GNS, and fertile SN (Halder et al., 2023; Wang et al., 2023; Qiao et al., 2022; Xu et al., 2022; Zhai et al., 2016). However, the two haplotypes identified at *QSl.gaas-5A* did not exhibit significant phenotypic differences, and the locus explained only 4% of the phenotypic variance. SL is a complex trait controlled by numerous minor-effect polygenes, and the contribution of any individual locus tends to be modest. This may account for the lack of significant haplotype effects observed at this locus.

The coincidence of several loci with those identified in earlier studies reflects the structural complexity and linkage relationships within the wheat genome, while also reinforcing the reliability of the association mapping results presented here. Although actual grain yield was not measured in this study, the yield components examined (SL, SNS, GNS, TKW) are well−established proxies for yield potential in wheat (Evans, 1993). The identified loci showed consistent effects across multiple environments and models, and their favorable haplotypes were associated with increased values of one or more yield components. Future studies using independent populations and actual yield data are warranted to further validate the breeding value of these loci.

## 5 Conclusions

This study evaluated 372 wheat landraces from Gansu Province for SL, SNS, GNS, and TKW across five environments. GWAS identified 19 loci associated with these yield-related traits. Comparative physical mapping suggested that *QSl.gaas-2D.1*, *QSl.gaas-2D.2*, *QSl.gaas-3B*, *QSl.gaas-4B*, *QSl.gaas-5B.1*, *QSl.gaas-7B*, *QGns.gaas-2A.2*, *QTkw.gaas-2D*, and *QTkw.gaas-4B* are likely novel loci with potential value for wheat breeding. A total of 16 candidate genes associated with spike traits were predicted. Haplotype analysis revealed the presence of favorable alleles in landraces conferring high spikelet density (i.e., short spikes with numerous spikelets), which could be exploited in breeding programs aimed at enhancing yield potential. This study provides newly identified loci and candidate gene resources for the genetic improvement of wheat spike architecture. 

## Data Availability

The original contributions presented in the study are included in the article/**Supplementary Material**. Further inquiries can be directed to the corresponding authors.

## Supplementary material

Supplementary Figure 1 Manhattan and Q-Q plots for SL. Images A to E represent Manhattan plots generated from genome-wide association studies (GWAS) for the spike length (SL) trait under different environmental conditions (24LZ,22QS, 23QS, 24QS, 25QS). The Manhattan plots from the center outward correspond to different models in sequence: BLINK, CMLM, FarmCPU, MLM, MLMM. The same as **Supplementary Figures 2**-**4**. Images F to J represent Quantile-quantile (Q-Q) plots generated from genome-wide association studies (GWAS) for the spike length (SL) trait under different environmental conditions (24LZ,22QS, 23QS, 24QS, 25QS).

Supplementary Figure 2 Manhattan and Q-Q plots for SNS. Images A to E represent Manhattan plots generated from genome-wide association studies (GWAS) for spikelet number per spike (SNS) trait under different environmental conditions (24LZ, 22QS, 23QS,24QS, 25QS). Images F to J represent Quantile-quantile (Q-Q) plots generated from genome-wide association studies (GWAS) for spikelet number per spike (SNS) trait under different environmental conditions (24LZ, 22QS, 23QS, 24QS, 25QS).

Supplementary Figure 3 Manhattan and Q-Q plots for GNS. Images A to E represent Manhattan plots generated from genome-wide association studies (GWAS) for grain number per spike (GNS) trait under different environmental conditions (24LZ, 22QS, 23QS, 24QS, 25QS). Images F to J represent Quantile-quantile (Q-Q) plots generated from genome-wide association studies (GWAS) for grain number per spike (GNS) trait under different environmental conditions (24LZ, 22QS, 23QS,24QS, 25QS).

Supplementary Figure 4 Manhattan and Q-Q plots for TKW. Images A to E represent Manhattan plots generated from genome-wide association studies (GWAS) for thousand-kernel weight (TKW) trait under different environmental conditions (24LZ, 22QS, 23QS,24QS, 25QS). Images F to J represent Quantile-quantile (Q-Q) plots generated from genome-wide association studies (GWAS) for thousand-kernel weight (TKW) trait under different environmental conditions (24LZ, 22QS, 23QS, 24QS, 25QS).

Supplementary Figure 5 Expression pattern analysis of candidate genes. The heatmap, based on the Hexaploid Wheat Expression Database (TPM values) from WheatOmics 1.0, illustrates the expression patterns of candidate genes across five wheat tissues (root, stem, leaf, spike, and grain). The expression data were row-wise Z-score normalized, with colors ranging from green (lower expression) to pink (higher expression) indicating relative expression levels. Hierarchical clustering of the candidate genes was performed using Euclidean distance. **(A)** Relative expression levels of candidate genes for spike length loci *QSl.gaas-2D.1* and *QSl.gaas-2D.2* in different tissues. **(B)** Relative expression levels of candidate genes for grain number per spike loci *QGns.gaas-2A.2* in different tissues. **(C)** Relative expression levels of candidate genes for thousand-kernel weight loci *QTkw.gaas-4B* in different tissues.

Supplementary Figure 6 Haplotype analysis of SL loci *QSl.gaas-3B, QSl.gaas-4B, QSl.gaas-5A, QSl.gaas-5B.1, QSl.gaas-5B.2, QSl.gaas-7B*. Note: MW, Mann‑Whitney U test; KW, Kruskal-Wallis test; p, p‑value); n, Sample size. The same as **Figures S7**-**9**.

Supplementary Figure 7 Haplotype analysis of SNS loci *QSns.gaas-7A*.

Supplementary Figure 8 Haplotype analysis of GNS loci *QGns.gaas-2A.1, QGns.gaas-2A.3, QGns.gaas-7A*.

Supplementary Figure 9 Haplotype analysis of TKW loci QTkw.gaas-1B, *QTkw.gaas-2D, QTkw.gaas-4B, QTkw.gaas-5B*.
