# Supplementary figures and images for: Uncovering yield-related traits loci in Chinese wheat landraces using GWAS
Source: Front Plant Sci. 2026 Jun 15;17:1838087. doi: 10.3389/fpls.2026.1838087 (PMC13311083; doi:10.3389/fpls.2026.1838087)

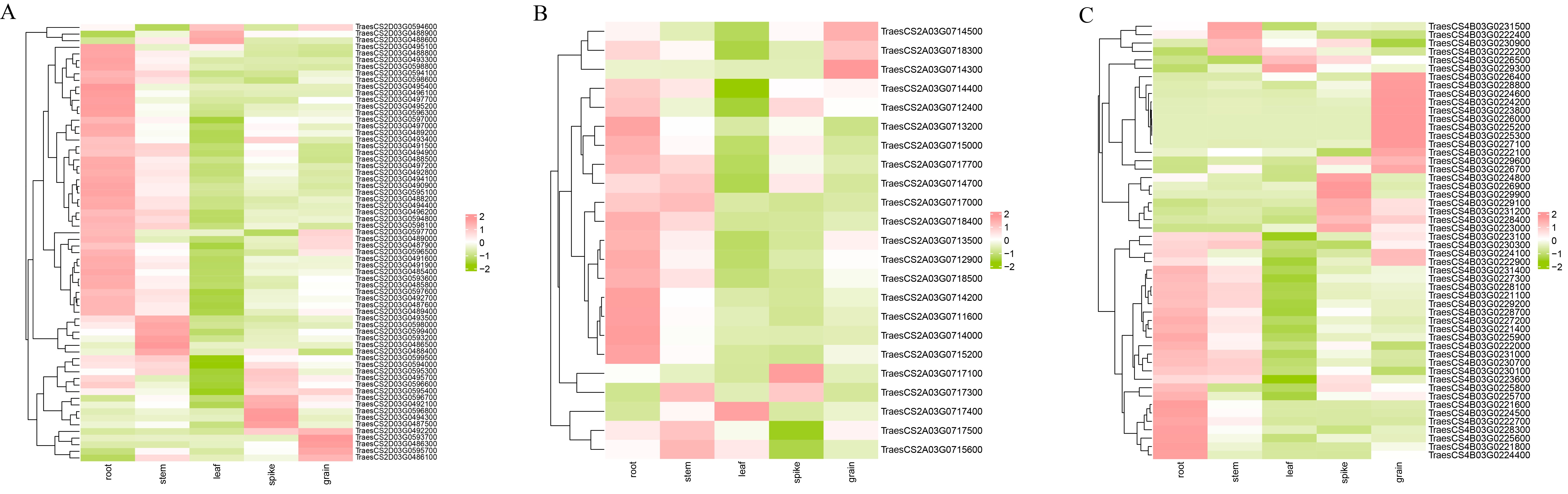

Supplement: Supplementary Figure 5 — Expression pattern analysis of candidate genes. The heatmap, based on the Hexaploid Wheat Expression Database (TPM values) from WheatOmics 1.0, illustrates the expression patterns of candidate genes across five wheat tissues (root, stem, leaf, spike, and grain). The expression data were row-wise Z-score normalized, with colors ranging from green (lower expression) to pink (higher expression) indicating relative expression levels. Hierarchical clustering of the candidate genes was performed using Euclidean distance. (A) Relative expression levels of candidate genes for spike length loci QSl.gaas-2D.1 and QSl.gaas-2D.2 in different tissues. (B) Relative expression levels of candidate genes for grain number per spike loci QGns.gaas-2A.2 in different tissues. (C) Relative expression levels of candidate genes for thousand-kernel weight loci QTkw.gaas-4B in different tissues. [file Image5.jpg]

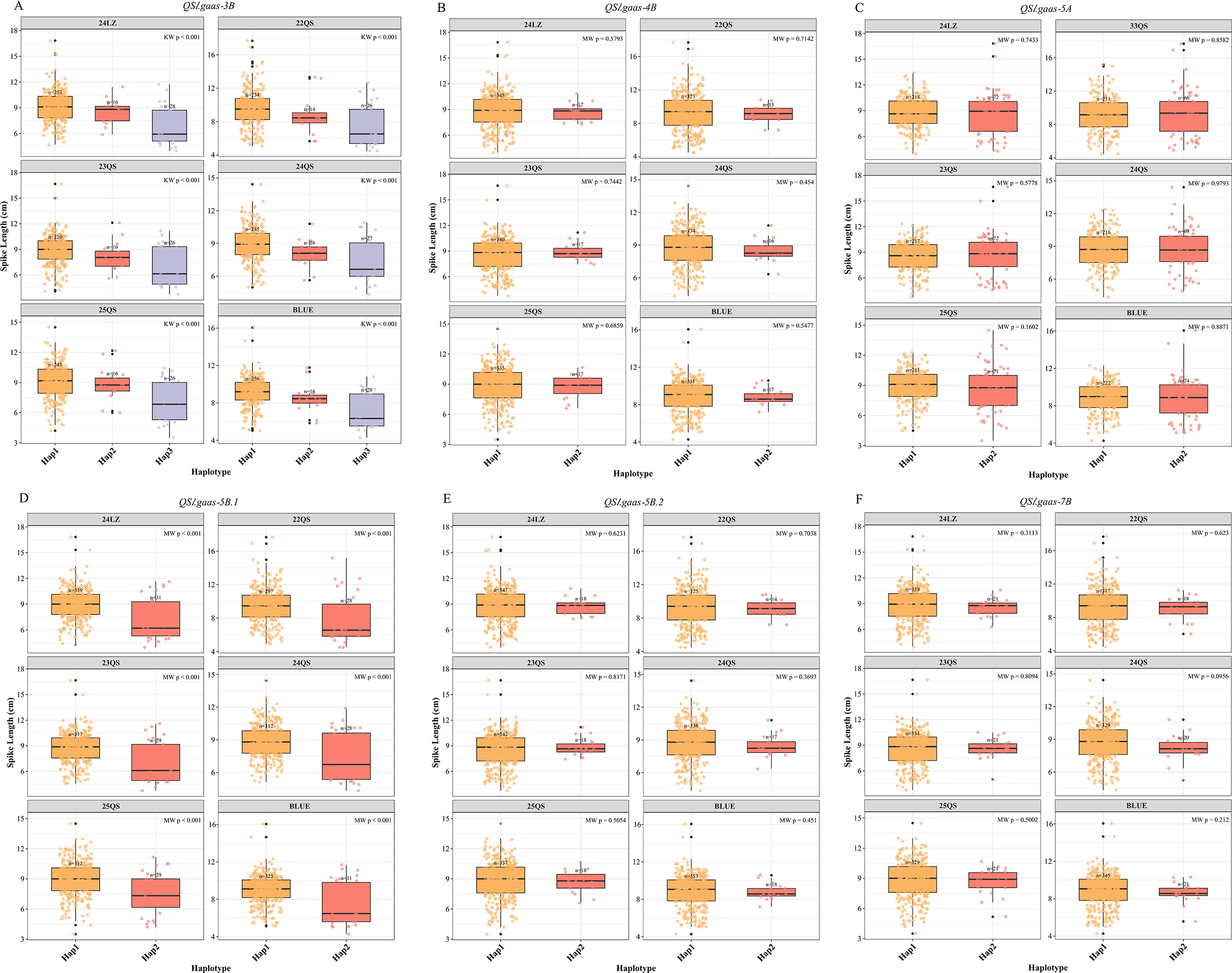

Supplement: Supplementary Figure 6 — Haplotype analysis of SL loci QSl.gaas-3B, QSl.gaas-4B, QSl.gaas-5A, QSl.gaas-5B.1, QSl.gaas-5B.2, QSl.gaas-7B. Note: MW, Mann‑Whitney U test; KW, Kruskal-Wallis test; p, p‑value); n, Sample size. The same as Figures S7-9. [file Image6.jpg]

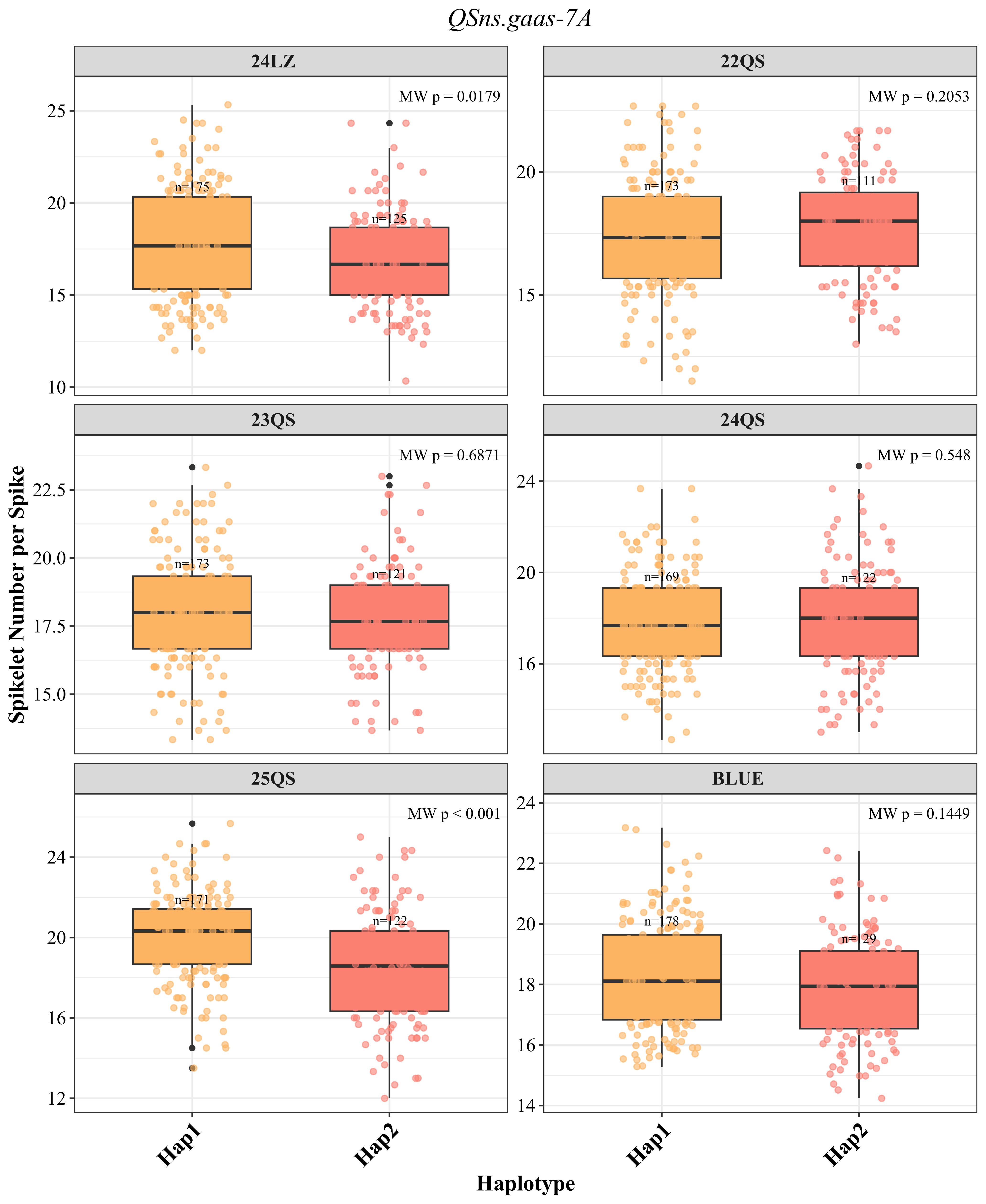

Supplement: Supplementary Figure 7 — Haplotype analysis of SNS loci QSns.gaas-7A. [file Image7.jpg]
